# Thermal behaviour of walnut shells by thermogravimetry with gas chromatography–mass spectrometry analysis

**DOI:** 10.1098/rsos.180331

**Published:** 2018-09-12

**Authors:** Fangyu Fan, Han Li, Yuqiao Xu, Yun Liu, Zhifeng Zheng, Huan Kan

**Affiliations:** 1 Key Laboratory for Forest Resources Conservation and Utilisation in the Southwest Mountains of China, Ministry of Education, Southwest Forestry University, 650224 Kunming, Yunnan, People's Republic of China; 2 School of Light Industry and Food Engineering, Southwest Forestry University, 650224 Kunming, People's Republic of China

**Keywords:** pyrolysis, walnut shell, DAEM, activation energy, TG-GC-MS, distributed activation energy model

## Abstract

The present study introduces thermogravimetry with gas chromatography–mass spectrometry (TG-GC-MS) at four different heating rates to investigate the activation energy and thermal degradation behaviour of walnut shell pyrolysis. The distributed activation energy model (DAEM) was applied to investigate the activation energy. According to values of the activation energy and the correlation coefficient by the DAEM, the activation energy (98.69–267.75 kJ mol^−1^) and correlation coefficient (0.914–0.999) were determined for pyrolysis of walnut shells. GC-MS was performed to investigate the pyrolysis products from walnut shells at different critical temperature points. More than 20 different substances were identified at different temperatures from GC-MS results. With the increasing pyrolysis temperature, furan, furfural, benzene and long chain alkanes were successively identified in different GC-MS experimental results.

## 1. Introduction

The growing worldwide demand for energy and continuous CO_2_ emissions have aroused the concern of researchers. Agricultural and forestry waste can serve as a rich source of lignocellulose feedstocks for biofuel production [1]. Comprehensive utilization of agricultural and forestry waste has become one of the factors restricting economic development. At present, incorrect treatment of this waste has led to serious environmental pollution [2]. In recent years, various researchers have studied the energy utilization of biomass waste and aimed to solve the current energy crisis and alleviate the environmental pollution caused by fossil energy combustion [3–5]. Pyrolysis is a commonly used thermochemical conversion technology for the preparation of gases, solids and liquids through biomass [6]. Most researchers produce biomass energy from agricultural and forestry waste by pyrolysis. China is a large agricultural country producing huge amounts of agricultural and forestry waste every year [7]. Biomass energy is produced from agricultural and forestry waste and has broad prospects in China. Walnut is widely planted in the world. In China, walnut planting areas have developed rapidly in recent years [8]. Much walnut shell waste, which contains abundant lignocellulose, will be produced in walnut processing, and can be used to produce diverse forms of energy by pyrolysis.

Thermogravimetric analysis (TG) has been widely used to analyse the thermodynamic behaviour of biomass decomposition under either inert gases or air. Thermogravimetric analysis–Fourier transform infrared (FTIR) was introduced to investigate the change rules of pyrolysis products during the pyrolytic process [9]. TG-FTIR technology can be used to analyse the functional groups of the escaping gas during weight loss of samples in TG. The gas composition can be analysed and assessed, and volatile gases can be quantitatively and qualitatively analysed [9–11]. TG coupled with mass spectrometry (TG-MS) is commonly used to investigate the release patterns and identities of volatile components in biomass pyrolysis and can be used to analyse the thermal decomposition and evolved gas characteristics of all kinds of biomass [12–14]. TG-FTIR and TG-MS are effective techniques used in the study of biomass pyrolysis. TG-FTIR can be used to detect functional group decomposition of biomass, whereas TG-MS can be used to analyse small molecules (H_2_, CH_4_, H_2_O, CO and CO_2_). However, identifying the compounds can be difficult owing to overlapping of numerous signals when the thermal degradation of the biomass produces a wide range of low-molecular-weight compounds given that separation of the gases that are released is not possible for TG-FTIR and TG-MS systems [15,16]. In addition, TG-FTIR can only be used to analyse the functional groups of products, but the specific products cannot be identified, and the analysis results are not accurate [9].

The TG-GC-MS system is another advanced method that can be used for analysis of volatile gases; it can be used to estimate product compositions when volatile gases are released at specific temperatures, contributing to our understanding of the pyrolytic process [15,17–19]. During TG-GC-MS experiments, GC-MS can be employed to separate and identify volatile components from samples under temperature conditions compatible with the TG experiments. Thus, TG-GC-MS results can provide a lot of information on volatile gas products and can be used to assess the reaction point of pyrolysis. He *et al*. [15] observed that results of TG-GC-MS presented mass loss and released gas information with the programmed temperature, improving the understanding of the structure of lignite and its pyrolytic process. Fedelich [17] analysed the quantification of low-content styrene–butadiene rubber in natural rubber by TG-GC-MS, and the results indicated that TG-GC-MS is a novel method that can be used to investigate the pyrolytic process. Feng *et al*. [18] evaluated the molecular structure of Shengli lignite by analysing the structure and content of the pyrolysis products via TG-GC-MS. With regard to the analytical methods, TG-GC-MS is similar to pyrolysis–gas chromatography–mass spectrometry (PY-GC-MS), which can be used to analyse the volatile components of pyrolysis under certain temperature conditions [20–22]. Compared with PY-GC-MS, TG-GC-MS results can provide much information and contribute to identifying the reaction point of pyrolysis [15]. With TG-GC-MS, the pyrolysis process of walnut shells can be analysed, and the production of the critical pyrolysis points can be more clearly understood, which provides more abundant data for the application of walnut shells. In general, the TG-GC-MS system is a novel technique that can provide more information for further understanding of the pyrolytic process.

In this study, TG-GC-MS is used to identify the thermal decomposition of walnut shells from room temperature to 900°C at a rate of 20°C min^−1^. TG-GC-MS can provide the necessary dynamics information to reveal pyrolysis mechanisms and to analyse the thermal behaviour of walnut shells. TG experiments on walnut shells were carried out at different heating rates to estimate the pyrolysis process. Moreover, pyrolysis activation energies were also studied by using the distributed activation energy model (DAEM) based on TG data.

## 2. Material and methods

### 2.1. Materials

The walnut shells originated from Yunnan Province, China. The walnut shells were washed with tap water and deionized water, and then dried at 105°C for 24 h. Dried walnut shells were crushed to less than 0.075 mm (approx. 200 mesh) for preparation of test samples. Table 1 shows the approximate and ultimate analysis results of the walnut shells. The approximate analysis was conducted using a 5E-MAG6600 Automatic Proximate Analyzer (China). The ultimate analysis (C, H, N and S) was performed using a Vario EL III Elemental Analyzer (Elementar, Germany) and the oxygen content was calculated on a dry ash-free base by difference.

**Table 1.** Properties of walnut shells.

approximate analysis (wt. %)^a^	
volatile matter	78.37
ash	3.05
fixed carbon	18.58
ultimate analysis (wt. %)^b^	
C	48.65
H	5.52
O^c^	42.14
N	0.49
S	0.15

^a^Dry base.

^b^Dry ash-free base.

^c^By difference.

### 2.2. Methods

The pyrolysis of walnut shells was analysed by TG (NETZSCH, Germany) within a temperature range of room temperature to 900°C at a heating rate of 10, 20, 40 and 60°C min^−1^ under a high-purity helium flow rate of 50 ml min^−1^. A total sample of 20.00 mg was pyrolyzed by TG.

The heating rate was chosen at 20°C min^−1^ during the TG-GC-MS experiment. Volatile gases at a target temperature were collected via an Automation Autoinjector™ system which was connected to the TG system. The quantitative loop was 1 ml, and the injection time was 10 s.

TG and GC-MS were connected by the transmission line at 300°C to prevent condensation of the pyrolysis gases during transmission. Gas analysis was estimated with a Thermo Scientific ISQ^TM^ quadrupole GC-MS system. After sample pyrolysis, pyrolysis gases were separated from the pipeline through GC and a chromatographic column. The separated substances were detected in the MS system. The chromatographic column was a TG-5MS (Thermo Scientific) capillary column (30 m length, 0.25 mm ID and 0.25 µm film). The oven temperature was held at 40°C for 1 min, increased from 40°C to 100°C at a rate of 1.5°C min^−1^ and held for 2 min, from 100°C to 150°C at a rate of 5°C min^−1^ and held for 1 min, and from 150°C to 200°C at a rate of 10°C min^−1^ and held for 1 min. Helium was used as the carrier gas for the column at a flow rate of 1.3 ml min^−1^. The detector consisted of a mass selective detector, and electron impact mass spectra were acquired with an ionizing energy of 70 eV with a scanning range from 50 to 600 *m*/*z* and a scan rate of 4 scans s^−1^. The MS transfer line and ion source temperature were maintained at 280°C.

### 2.3. Analysis of activation energy

In this study, the DAEM was selected to calculate the activation energy. Analysis of the literature showed that the DAEM can be feasibly used to analyse biomass kinetics and coal pyrolysis [23–25]. The DAEM assumes that all reaction energies possess the same *k*_0_ at the same conversion rate (mass fraction at time *t* or temperature *T*), whereas the activation energy shows a continuous distribution. The DAEM is represented as follows [23]:

2.1 $$\frac{V}{V^{*}}=1-\int_{0}^{\infty }\exp\left[-\frac{k_{0}}{\beta }\int_{0}^{T}\exp\left(-\frac{E}{RT}\right)dT\right]f\;(E)dE,$$

where *V* denotes the volatile content at temperature *T*, V* refers to the effective volatile content of the sample, *k*_0_ is the frequency factor corresponding to the *E* value, *β* represents the heating rate and *f*(*E*) corresponds to the distribution curve of the activation energy representing the difference in activation energies of various first-order irreversible reactions [23,26]. The equation was simplified by Miura [27] as follows:

2.2 $$\frac{V}{V^{*}}=1-\int_{E_{s}}^{\infty }\;f\;(E)\text{d}E=\int_{0}^{E_{s}}\;f\;(E)\text{d}E,$$

where *E*_s_ is the activation energy at a given temperature. In this simplified model, the Arrhenius equation can be described as follows [27,28]:

2.3 $$\text{ln}\left(\frac{\beta }{T^{2}}\right)=\text{ln}\left(\frac{k_{0}\text{R}}{E}\right)+0.6075-\frac{E}{\text{R}T}.$$

Both *E* and *k*_0_ can be calculated from the slope and intercept of the Arrhenius plot, respectively.

## 3. Results and discussion

### 3.1. Thermogravimetric analysis

**Figure 1.**
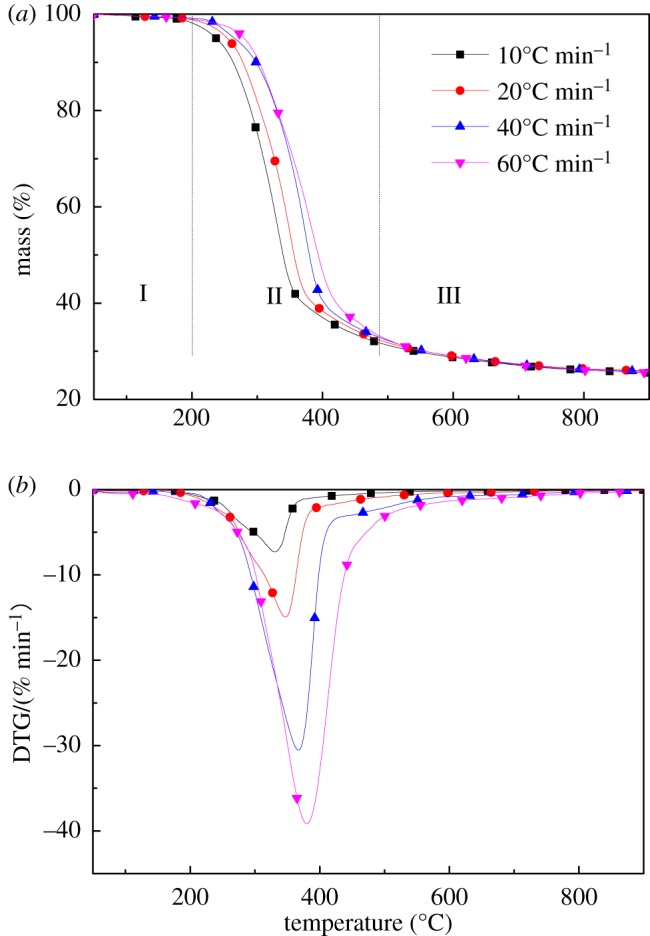
TG (*a*) and DTG (*b*) curves of walnut shell pyrolysis at different heating rates.

Figure 1*a* presents the TG and derivative thermogravimetry (DTG) curves with different heating rates ranging from 50°C to 900°C. As lignocellulose is stable at 200°C [29], mass loss is minimal, and its removal mainly occurs as a result of a small amount of moisture and pyrolysis of some extractives. Some researchers observed two remarkable peaks in biomass pyrolysis [30]; this result is attributed to the presence of more water and extractive content than walnut shells. Pyrolysis was analysed at 20°C min^−1^ as an example. It can be seen from figure 1*a* that about 90% weight loss occurred in the temperature range from 200°C to 467°C. Pyrolysis of walnut shells can be divided into three stages. The first stage (I) represents the weight loss due to water content and extractives. The second stage (II) refers to the rapid thermal decomposition between 200°C and 500°C and corresponds to pyrolysis of cellulose, hemicellulose and a part of lignin [31]. Lignin is mainly pyrolyzed at temperatures above 400°C. The pyrolysis products of cellulose and hemicellulose mainly consist of gases; thus, mass loss is large and pyrolysis occurs rapidly. On the contrary, the pyrolysis products of lignin are dominated by solids; thus, the mass change is small and pyrolysis proceeds slowly. The last stage (III) is high-temperature charring of the residue and weight loss is much smaller [22].

It can be seen from figure 1*b* that DTG curves for walnut shells at different heating rates showed a similar mass loss behaviour, exhibiting a single peak at 330°C, 347°C, 367°C and 385°C. With increasing heating rate, pyrolysis leads to an increased maximum mass loss rate and temperature of the walnut shells. The maximum mass loss rate of walnut shells was 7.25% min^−1^, 14.95% min^−1^, 31.25% min^−1^ and 38.5% min^−1^ at heating rates of 10°C, 20°C, 40°C and 60°C min^−1^, respectively. The reason for this is that there is insufficient time available for heat transfer within the biomass at high heating rates, leading to a non-homogeneous temperature and reaction distribution, and, thus, the peak shifts in the DTG curves [32].

### 3.2. Activation energy calculated from the distributed activation energy model

**Figure 2.**
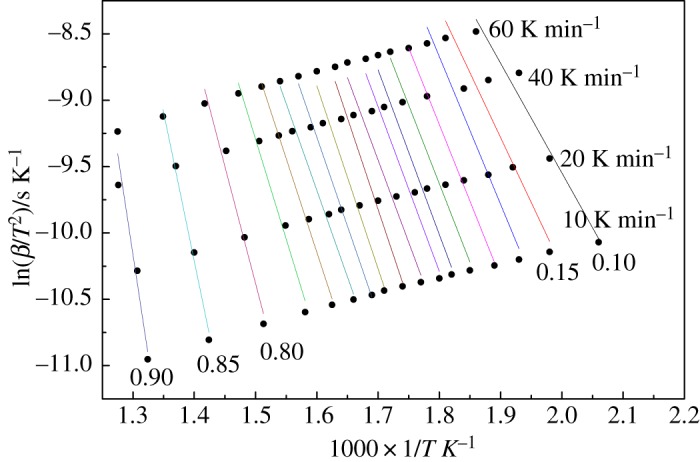
Parameters of the DAEM for walnut shells.

**Figure 3.**
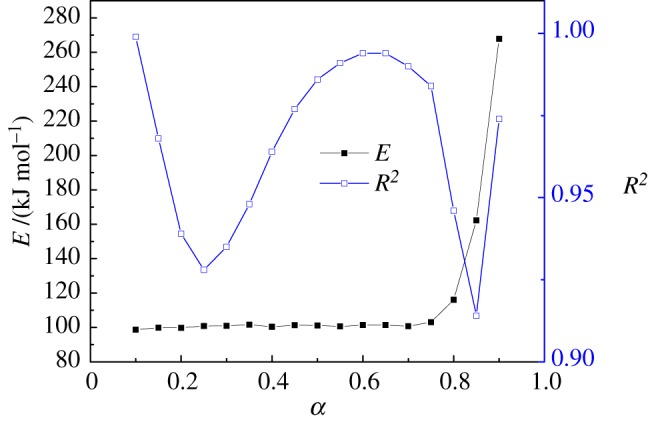
Activation energy and correlation factor from the DAEM for walnut shells.

The Arrhenius plot of ln (*β*/*T*^2^) and 1/*T* is shown in figure 2. The linear and parallel development for different conversion rates ranged from 0.10 to 0.90 at different heating rates. Results indicate that pyrolysis of walnut shells can be described by a set of similar single reactions. Equation (2.3) was used to calculate activation energies and the correlation coefficient at each conversion rate from the Arrhenius plot, and the results are shown in figure 3. Activation energies changed slightly from 98.69 kJ mol^−1^ to 116.08 kJ mol^−1^, corresponding to the conversion rates from 0.1 to 0.8. At a conversion rate of 0.8–0.9, the activation energies changed remarkably from 162.24 to 267.75 kJ mol^−1^. The effect of heat and mass transfer on the pyrolysis reaction result is assumed to cause difficulties in the reaction, and activation energies are higher at the end of the pyrolysis reaction. A number of researchers [25,26] have observed similar phenomena for biomass pyrolysis. With regard to the correlation coefficient, values ranged from 0.914 to 0.999, indicating the reliability of the analysis results of the kinetics. Compared with the research results of Açikalin [33], the pyrolysis activation energy of walnut shells was higher in the present study. The reason for this is that the activation energy is calculated using different conversion ratios, while the average activation energy was studied in the research of Açikalin.

### 3.3. Gas chromatography–mass spectrometry series analysis

During pyrolysis, products are continually formed; analysis of the pyrolysis products at each time point becomes impossible when using GC-MS. Pyrolysis mass loss at the low-temperature stage is attributed to water content and extractive pyrolysis [34]. The 300–400°C stage is the main pyrolysis stage of biomass, and various products are formed at this stage [35]. In the last stage, the main processes comprise the final stage of lignin pyrolysis and secondary reactions of the residue [36,37]. Critical temperature points were selected at mass losses of 10%, 90%, 98% and the maximum weight loss rate, and temperature points measured at 268°C, 467°C, 695°C and 347°C, respectively.

**Figure 4.**
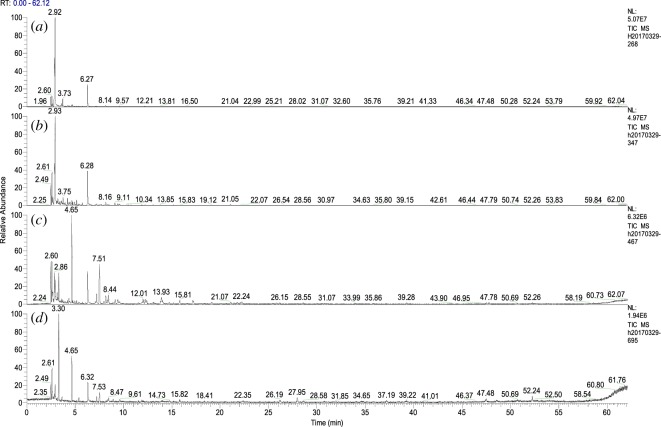
GC-MS spectrum for pyrolysis products evolving from walnut shells at different temperatures. (*a*) 268°C, (*b*) 347°C, (*c*) 467°C and (*d*) 695°C.

Figure 4 and table 2 show the GC-MS spectra and product components of walnut shells at different pyrolysis temperatures, respectively. A matching degree higher than 75% was accepted in detecting products except for the last four products in table 2. The last four products have a matching degree of 60–65%. Figure 4 shows the complex pyrolysis products at each temperature point. Table 2 lists the components detected by GC-MS at different pyrolysis temperatures.

**Table 2.** Analysis of the main pyrolysis components by GC-MS of walnut shells at different temperatures.

retention time (min)	name	chemical formula	temperature (°C)			
			268	347	467	695
2.47	2-butene	C_4_H_8_	+	+	+	+
2.61	*sec*-butylamine	C_4_H_11_N	+	+	+	+
2.7	1,3-cyclopentadiene	C_5_H_6_	−	+	+	−
2.86	urea	CH_4_N_2_O	+	+	+	−
2.92	furan, 2-methyl-	C_5_H_6_O	+	+	+	+
3.15	1,4-cyclohexadiene	C_6_H_8_	−	−	+	−
3.20	2-butenal	C_4_H_6_O	−	+	−	−
3.32	benzene	C_6_H_6_	−	−	+	+
3.65	heptane	C_7_H_16_	−	−	−	+
3.75	furan, 2,5-dimethyl-	C_6_H_8_O_2_	+	+	−	−
4.19	1H-pyrrole,1-methyl-	C_5_H_7_N	−	+	−	−
4.26	disulfide, dimethyl	C_2_H_6_S_2_	+	+	−	−
4.66	toluene	C_7_H_8_	−	+	+	+
5.15	cyclopentanone	C_5_H_8_O	−	+	−	−
5.39	hexane, 3-ethyl-	C_8_H_18_	−	−	−	+
6.29	furfural	C_5_H_4_O_2_	+	+	+	+
7.23	ethylbenzene	C_8_H_10_	−	−	+	+
7.53	benzene,1,3-dimethyl-	C_8_H_10_	−	−	+	+
8.17	4-cyclopentene-1,3-dione	C_5_H_4_O_2_	−	+	+	−
8.39	*O*-xylene	C_8_H_10_	−	−	+	+
9.12	2-cyclopenten-1-one, 2-methyl-	C_6_H_8_O	−	−	+	−
12.01	benzene,1-ethyl-4-methyl-	C_9_H_12_	−	−	+	−
13.93	benzene, 1,2,4-trimethyl-	C_9_H_12_	−	−	+	−
15.85	benzene,1-methoxy-3-methyl-	C_8_H_10_O	−	−	+	−
27.95	1H-indene,1-methylene-	C_10_H_8_	−	−	−	+
39.22	tridecane	C_13_H_28_	−	−	−	+
47.48	decane, 2-methyl-	C_11_H_24_	−	−	−	+
52.24	pentadecane, 7-methyl-	C_16_H_34_	−	−	−	+

‘+' Indicates the presence of substance and ‘−'indicates the absence of substance.

It can be seen from table 1 that the pyrolysis products of walnut shells escaped at different pyrolysis temperatures, and the products differed in composition at different temperatures. Several simple products were observed at 268°C. However, the products exhibited complexity and diversity at 467°C. At a high pyrolysis temperature (695°C), a variety of complex products were produced owing to pyrolysis of lignin and the occurrence of secondary reactions [37,38].

During pyrolysis at 268°C, the pyrolysis products of walnut shells mainly included low-carbon-number compounds, including 2-butene, 1,3-cyclopentadiene, furan, 2-methyl- and furfural. At the same time, the products showed that nitrogen- and sulfur-containing compounds in walnut shells have been pyrolyzed as proven by the detected urea, disulfide and dimethyl compounds. The products contained 2-butene and furfural, indicating that hemicellulose and cellulose were pyrolyzed, and the unstable branched chain disintegrated [38,39]. The pyrolysis temperature of hemicellulose is low, and hemicellulose starts to decompose at 200°C, and the volatile matter split over rapidly at 200°C–400°C. The pyrolysis temperature of cellulose is about 270°C–420°C, and the pyrolysis temperature is higher than that for hemicellulose [40]. Pyrolysis products indicated that the pyrolysis of hemicellulose is dominant at 268°C.

At 347°C, the maximum mass loss rate of walnut shells reached 14.95% · min^−1^, and large amounts of pyrolysis products were detected; these products included furan, 2-methyl-, furfural, 2-butenal, furan and 2,5-dimethyl- compounds. Concerning the quantity and distribution of products, pyrolysis products at 347°C were simple, but amounts were increased. Table 2 shows that the compounds mainly consisted of short chains (C4 and C5). This phenomenon was attributed to the destruction of the polymerized structure of cellulose and hemicellulose at 347°C [36]. Toluene was detected in the product, indicating that lignin was pyrolyzed [41].

At 467°C, the pyrolytic products are complex and diverse. This is due to the combined effects of pyrolysis products at 467°C and in the previous pyrolysis process. As pyrolysis is 90% complete, cellulose and hemicellulose were completely pyrolyzed. In this process, lignin was mainly pyrolyzed, and the products contained more benzene compounds, such as ethylbenzene, 1,3-dimethyl-benzene, and so on. Compared with the pyrolysis process of cellulose and hemicellulose, the pyrolysis temperature of lignin is wide, ranging from 200°C to 500°C.

At 695°C, the mass is reduced by 98%, and the pyrolysis reaction ended. The products contained high amounts of benzene-containing compounds. Meanwhile numerous long-chain hydrocarbons were also detected because of pyrolysis of the residue, resulting in the production of long-chain hydrocarbons.

## 4. Conclusion

This study aimed to investigate pyrolysis of walnut shells, and analysed the activation energies and thermal degradation behaviour by TG-GC-MS. The DAEM was applied to investigate the reaction activation energy. The activation energies changed slightly from 98.69 to 116.08 kJ mol^−1^, corresponding to conversion rates from 0.1 to 0.8. This TG-GC-MS research investigated the pyrolysis products from walnut shells at different critical temperature points. More than 20 different substances were identified at different temperatures from GC-MS results. Furan, furfural, benzene and long-chain alkanes were successively identified in different GC-MS experimental results with increasing pyrolysis temperature.

## Data accessibility

This article does not contain any additional data.
